# Increased Life Span due to Calorie Restriction in Respiratory-Deficient Yeast

**DOI:** 10.1371/journal.pgen.0010069

**Published:** 2005-11-25

**Authors:** Matt Kaeberlein, Di Hu, Emily O Kerr, Mitsuhiro Tsuchiya, Eric A Westman, Nick Dang, Stanley Fields, Brian K Kennedy

**Affiliations:** 1 Departments of Genome Sciences and Medicine, University of Washington, Seattle, Washington, United States of America; 2 Department of Biochemistry, University of Washington, Seattle, Washington, United States of America; 3 Howard Hughes Medical Institute, University of Washington, Seattle, Washington, United States of America; Stanford University School of Medicine, United States of America

## Abstract

A model for replicative life span extension by calorie restriction (CR) in yeast has been proposed whereby reduced glucose in the growth medium leads to activation of the NAD^+^–dependent histone deacetylase Sir2. One mechanism proposed for this putative activation of Sir2 is that CR enhances the rate of respiration, in turn leading to altered levels of NAD^+^ or NADH, and ultimately resulting in enhanced Sir2 activity. An alternative mechanism has been proposed in which CR decreases levels of the Sir2 inhibitor nicotinamide through increased expression of the gene coding for nicotinamidase, *PNC1*. We have previously reported that life span extension by CR is not dependent on Sir2 in the long-lived BY4742 strain background. Here we have determined the requirement for respiration and the effect of nicotinamide levels on life span extension by CR. We find that CR confers robust life span extension in respiratory-deficient cells independent of strain background, and moreover, suppresses the premature mortality associated with loss of mitochondrial DNA in the short-lived PSY316 strain. Addition of nicotinamide to the medium dramatically shortens the life span of wild type cells, due to inhibition of Sir2. However, even in cells lacking both Sir2 and the replication fork block protein Fob1, nicotinamide partially prevents life span extension by CR. These findings (1) demonstrate that respiration is not required for the longevity benefits of CR in yeast, (2) show that nicotinamide inhibits life span extension by CR through a Sir2-independent mechanism, and (3) suggest that CR acts through a conserved, Sir2-independent mechanism in both PSY316 and BY4742.

## Synopsis

Calorie restriction slows aging and increases life span in nearly every organism studied. The mechanism by which this occurs is one of the most important unanswered questions in biogerontology. One popular theory, based on work from the budding yeast *Saccharomyces cerevisiae,* proposes that calorie restriction works by causing a metabolic shift toward increased mitochondrial respiration, resulting in activation of a family of proteins known as Sirtuins. This study demonstrates that life span extension by calorie restriction does not require respiration and occurs even in cells completely lacking mitochondrial DNA. Interestingly, calorie restriction protects yeast cells against a severe longevity defect associated with absence of mitochondrial DNA, suggesting the possibility that the consequences of age-associated mitochondrial dysfunction might be alleviated or prevented by calorie restriction.

## Introduction

Calorie restriction (CR) has been shown to slow aging in evolutionarily divergent species, including yeast, worms, flies, and rodents [1–5]. In addition to increasing longevity, CR is reported to cause additional phenotypes, including increased resistance to oxidative stress [6–8], enhanced DNA damage repair [9,10], decreased levels of oxidatively damaged proteins [11–13], improved glucose homeostasis and insulin sensitivity [14–16], altered levels of apoptosis [17], and delayed onset of a number of age-related diseases [18–21]. Although it has been known for more than 70 y that calorie restriction can increase life span in mammals [22], a mechanistic understanding of this phenomenon has remained elusive. It seems clear that nutrient and growth factor responsive pathways, such as those mediated by insulin, IGF-1, TOR, and Akt, are likely to represent important conduits through which these signals affect the aging rate. CR mediates enhancement of stress response pathways in mammals [23,24], and signaling through the insulin-like pathway in worms coordinates expression of a variety of antioxidant, chaperone, and anti-bacterial stress response proteins [25–27]. Similarly, TOR-mediated regulation of translational machinery appears to play a role in the response to nutrient deprivation in yeast [28], worms [29,30], flies [31], and mammals [32]. Finally, models postulating a role for Sir2-like protein deacetylases in CR-mediated life span extension have gained popularity for yeast [33], flies [34], and mammals, as well [4,35].

In the budding yeast *Saccharomyces cerevisiae,* CR can be imposed by reducing the concentration of glucose in the growth medium, resulting in a 20%–40% increase in replicative life span in multiple strain backgrounds [33,36,37]. In addition, genetic models of CR include deletion of the gene coding for hexokinase, *HXK2,* and mutations that decrease signaling through the cAMP-dependent protein kinase, PKA, such as deletion of the genes coding for the glucose sensing proteins *GPA2* or *GPR1,* and temperature-sensitive alleles of adenylate cyclase *(cdc35–1)* or the RAS-associated GTPase *(cdc25–10)* [33].

CR has been proposed to increase yeast replicative life span by a mechanism involving activation of Sir2 [33], an NAD^+^–dependent histone deacetylase [38–40] that inhibits the formation of extrachromosomal rDNA circles (ERCs) [41]. ERCs are self-replicating DNA molecules that accumulate in the mother-cell nucleus with age and are thought to cause senescence [42]. Overexpression of Sir2 increases life span in multiple strain backgrounds [36,41,43], and deletion of Sir2 shortens life span by about 50% [41,44]. CR fails to increase the life span of short-lived *sir2*Δ cells [33], consistent with the idea that CR could be acting by a mechanism involving Sir2.

The question of how CR might activate Sir2 has been a source of considerable controversy [45]. *Saccharomyces cerevisiae* is a facultative anaerobe that, under standard laboratory growth conditions (2% glucose), generates ATP largely by fermentation. Under conditions of reduced glucose, such as CR, *S. cerevisiae* shifts from fermentation to respiration, resulting in increased transcription of respiratory genes and a higher rate of oxygen consumption [46]. In models put forth by Lin et al., this metabolic shift results in activation of Sir2, either through increased cellular NAD^+^ [46] or decreased cellular NADH [47]. Alternatively, Anderson et al. have reported that CR does not alter NAD^+^ levels [48], but leads to enhanced expression of *PNC1* and a reduction in cellular nicotinamide [49]. Since nicotinamide is an inhibitor of the Sir2 deacetylation reaction, its decreased concentration could result in enhanced Sir2 activity [50,51]. Overexpression of Pnc1 suppresses the effect of exogenously added nicotinamide on Sir2-dependent silencing at HM loci, telomeres, and rDNA [52]; there are conflicting reports, however, on whether Pnc1 overexpression alters Sir2 activity at endogenous levels of nicotinamide [49,52].

More recently, we have questioned the importance of Sir2 in life span extension by CR [28,53]. In a long-lived strain background, BY4742, CR increases life span to a greater extent in cells lacking both Sir2 and the replication fork barrier protein Fob1 than in wild-type cells [36]. Based on this observation, and the fact that deletion of *SIR2* shortens life span by approximately 50%, we proposed a model whereby the inability of CR to increase life span in *sir2*Δ *FOB1* cells is explained as an indirect effect, resulting from the hyperaccumulation of ERCs [36]. Deletion of *FOB1* in a *sir2*Δ background suppresses the hyperaccumulation of ERCs, as well as the longevity defect [41], thus allowing CR to dramatically increase the life span of cells in the absence of Sir2 [36].

Much of the early work suggesting a link between CR and Sir2 was carried out in the PSY316 strain background [33,37,46,47,49,50,54], a strain in which, paradoxically, overexpression of Sir2 fails to increase life span [55]. Guarente and Picard [35] have proposed that CR might act via different mechanisms in the BY4742 and PSY316 strain backgrounds. In order to further clarify the molecular mechanism(s) underlying replicative life span extension by calorie restriction in yeast, we have sought to directly test key components of the models for Sir2-dependent CR in both of these strains. Here, we examine in detail the role of respiratory metabolism in life span extension by CR, finding that (1) respiration is not required for life span extension by CR; and (2) CR suppresses the enhanced early mortality, only apparent in PSY316, due to loss of mitochondrial DNA. In contrast, exogenous addition of nicotinamide partially, but not completely, blocks Sir2-independent life span extension by CR.

## Results

### Life Span Extension by CR Is Independent of Respiration in BY4742

**Figure 1:**
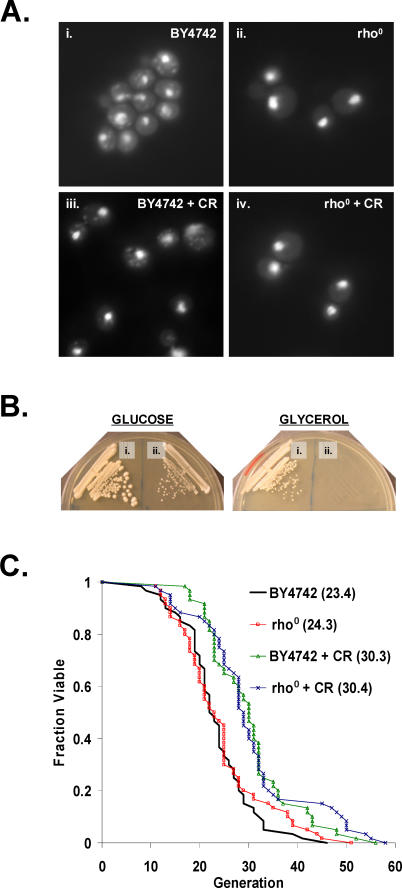
Respiration Is Not Required for Life Span Extension by CR in BY4742 (A) BY4742 rho^0^ strains lack mitochondrial DNA. DAPI staining of BY4742 (i) wild-type or (ii) rho^0^ cells grown under standard conditions (2% glucose) and calorie-restricted (iii) wild-type or (iv) rho^0^ cells (CR = 0.05% glucose). (B) BY4742 rho^0^ strains are unable to grow on glycerol as the sole carbon source. (i) BY4742 wild-type or (ii) rho^0^ cells on YEP supplemented with 2% glucose or 3% glycerol. (C) CR increase life span in BY4742 rho^0^ cells. Replicative life span analysis for BY4742 wild-type and rho^0^ cells on 2% glucose and 0.05% glucose (CR). Mean life span is shown in parentheses.

A central facet of the Sir2-dependent models for life span extension by CR is that a metabolic shift from fermentation to respiration in response to CR results in activation of Sir2 [46,47]. Since Sir2 is not required for life span extension by CR in BY4742 [36], we wished to determine whether respiration was also dispensable. The effect of CR in the absence of respiratory capacity was examined using rho^0^ cells, which completely lack mitochondrial DNA. Rho^0^ yeast lack three cytochrome c oxidase subunits *(COX1, COX2,* and *COX3)*, three ATP synthase subunits *(ATP6, ATP8,* and *ATP9)*, and apocytochrome b *(CYTB)*, and are therefore incapable of respiratory metabolism [56,57]. BY4742 rho^0^ cells were generated (see Materials and Methods), and the absence of mitochondrial DNA was verified by staining cells with DAPI (Figure 1A). Lack of respiratory capacity in rho^0^ cells was confirmed by inability to grow on the non-fermentable carbon source, glycerol (Figure 1B). As previously observed [58], replicative life span under standard conditions (2% glucose) was not altered by loss of mitochondrial DNA in this strain (Figure 1C). CR (0.05% glucose) significantly enhanced the life span of both wild-type and rho^0^ cells to a comparable degree. Thus, respiration is not required for life span extension by CR in the long-lived BY4742 strain background.

### Life Span Extension by CR Is Independent of Respiration in PSY316

**Figure 2:**
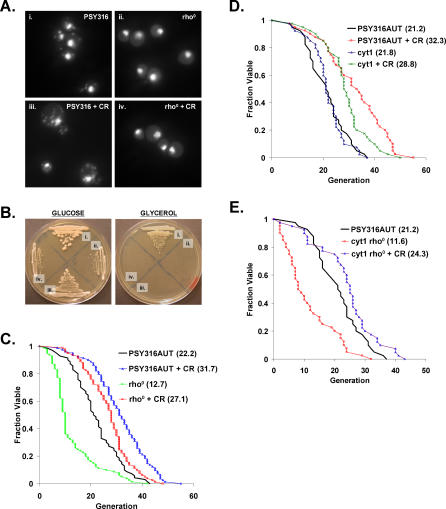
Respiration Is Not Required for Life Span Extension by CR in PSY316 (A) PSY316AUT rho^0^ strains lack mitochondrial DNA. DAPI staining of PSY316 (i) wild-type or (ii) rho^0^ cells grown under standard conditions (2% glucose) and calorie-restricted (iii) wild-type or (iv) rho^0^ cells (CR = 0.05% glucose). (B) PSY316AUT rho^0^ strains are unable to grow on glycerol as the sole carbon source. (i) PSY316AUT wild-type, (ii) *cyt1*Δ rho^0^, (iii) *cyt1*Δ*,* or (iv) rho^0^ cells on YEP supplemented with 2% glucose or 3% glycerol. (C) CR increases life span in PSY316AUT rho^0^ cells. Replicative life span analysis for PSY316AUT wild-type and rho^0^ cells on 2% glucose and 0.05% glucose (CR). Mean life span is shown in parentheses. (D) CR increases the life span of *cyt1*Δ cells. Replicative life span analysis for PSY316AUT wild-type and *cyt1*Δ cells on 2% glucose and 0.05% glucose (CR). Mean life span is shown in parentheses. (E) CR increases the life span of *cyt1*Δ rho^0^ cells. Replicative life span analysis for PSY316AUT wild-type and *cyt1*Δ rho^0^ cells on 2% glucose and *cyt1*Δ rho^0^ cells on 0.05% glucose (CR). Mean life span is shown in parentheses.

Previous work indicating that life span extension by CR is dependent on respiration was carried out in the PSY316 strain background, in which it was reported that deletion of the gene coding for cytochrome c1, *CYT1,* prevents life span extension by CR [46]. In order to address whether life span extension by CR in mutants incapable of respiratory metabolism is specific to BY4742, we generated respiratory-deficient *cyt1*Δ and rho^0^ variants in the PSY316 background (Figure 2A and 2B) and measured life span on medium containing either 2% or 0.05% glucose. As in BY4742 rho^0^ cells, CR significantly increased both mean and maximum life span in PSY316 rho^0^ (Figure 2C) and *cyt1*Δ cells (Figure 2D).

Unlike the case in BY4742 in which deletion of mitochondrial DNA has no effect on life span, PSY316 rho^0^ variants exhibited a profound increase in early mortality under standard growth conditions. This phenotype was not observed in *cyt1*Δ cells, indicating that loss of mitochondrial DNA, rather than general respiratory deficiency, is responsible for the life span defect. Deletion of *CYT1* in cells lacking mitochondrial DNA, however, resulted in a short life span comparable to that of rho^0^ cells (Figure 2E), demonstrating that rho^0^ is epistatic to *cyt1*Δ for this phenotype. As observed for rho^0^ cells, CR more than doubled the short life span of *cyt1*Δ rho^0^ cells, which contain both nuclear and mitochondrial mutations that prevent respiration.

**Figure 3:**
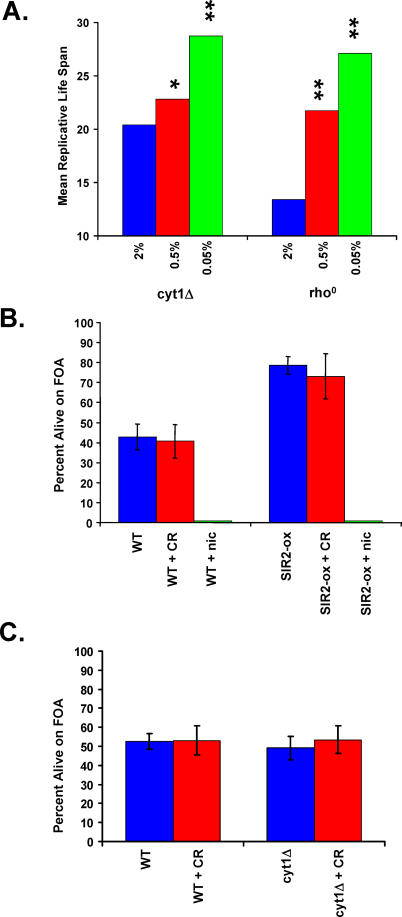
Effect of Glucose Concentration on Life Span and Sir2 Activity in Respiratory-Deficient Mutants (A) Mean replicative life span is significantly increased in PSY316 AUT *cyt1*Δ and rho^0^ cells as the glucose concentration is decreased to either 0.5% or 0.05%, relative to life span on 2% glucose. *p < 0.05, **p < 0.01. (B) Sir2 activity is not increased by CR but is responsive to increased expression of Sir2 or to addition of exogenous nicotinamide. Transcriptional silencing of the telomeric *URA3* marker in PSY316AUT (WT) was monitored by the survival of cells plated onto medium containing 5-FOA. (C) Sir2 activity is not altered in respiratory deficient *cyt1*Δ cells and is unaffected by CR. Transcriptional silencing of the telomeric *URA3* marker in PSY316AUT (WT) was monitored by the survival of cells plated onto medium containing 5-FOA.

Our observation that CR increased life span in PSY316 cells lacking *CYT1* is in contrast to a previous report [46]. A potential explanation for this difference is that 0.5% glucose was used for CR in the prior study, rather than the 0.05% glucose concentration used in this study. We therefore measured the life span of respiratory deficient rho^0^ and *cyt1*Δ cells grown on 0.5% glucose. As we observed for cells grown on 0.05% glucose, growth on 0.5% glucose increased the life span of rho^0^ and *cyt1*Δ cells, although the magnitude of life span extension is reduced relative to 0.05% glucose (Figure 3A). Thus, the use of a non-optimal level of CR may have precluded detection of life span extension by CR in *cyt1*Δ mutants in the prior study.

We also examined the effect of CR on Sir2 activity in respiratory-deficient PSY316 cells. The PSY316AUT variant has both *URA3* and *ADE2* marker genes integrated near telomeres, thus allowing for efficient determination of Sir2-dependent telomeric silencing in response to genetic or environmental perturbations [55]. As previously reported, increased Sir2 activity can be achieved by overexpression of *SIR2* in the PSY316 strain background [55], significantly enhancing telomere silencing and survival on FOA, while inhibition of Sir2 by addition of 5 mM nicotinamide to the medium decreased telomere silencing (Figure 3B). CR, however, had no detectable effect on Sir2-dependent silencing at telomeres. Similarly, respiratory deficiency caused by deletion of *CYT1* also fails to impact Sir2-dependent silencing at either 2% or 0.05% glucose (Figure 3C). A decrease in survival on FOA was observed in rho^0^ cells relative to wild-type or *cyt1*Δ cells at 2% glucose (Figure S1); however, CR failed to result in a detectable increase in Sir2 activity in respiratory deficient or respiratory competent cells. Therefore, we find no evidence that a metabolic shift from fermentation toward respiration is involved in life span extension by CR or that Sir2 is activated in response to CR.

### Nicotinamide Blocks Life Span Extension by CR

**Figure 4:**
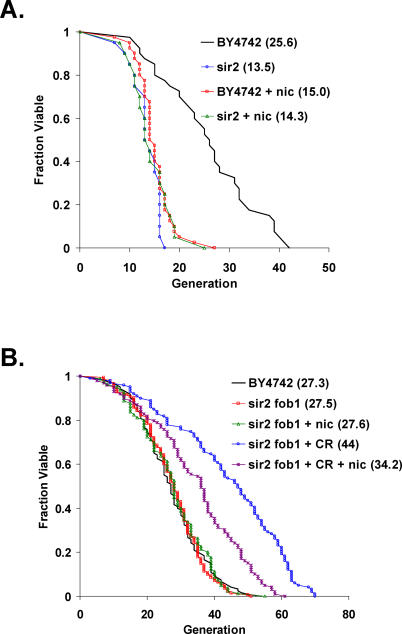
Effect of Nicotinamide on Life Span Extension by CR (A) Nicotinamide shortens the life span of wild-type cells. Replicative life span analysis for BY4742 wild-type and *sir2*Δ cells on 2% glucose containing or lacking 5 mM nicotinamide (nic). Mean life span is shown in parentheses. (B) Nicotinamide partially prevents Sir2-independent life span extension by CR. Replicative life span analysis for BY4742 wild type on 2% glucose, along with *sir2*Δ *fob1*Δ double mutant cells on 2% glucose or 0.05% glucose (CR) containing or lacking 5 mM nicotinamide (nic). Mean life span is shown in parentheses.

CR has also been proposed to activate Sir2 by reducing cellular pools of nicotinamide, a Sir2 inhibitor [50]. Addition of 5 mM nicotinamide to the medium prevents life span extension by CR in wild-type mother cells [49]; however, interpretation of this experiment is complicated by the fact that, like deletion of *SIR2*, high levels of nicotinamide result in a dramatically shortened life span. We took advantage of the fact that deletion of *FOB1* suppresses the short life span and ERC hyperaccumulation phenotypes associated with deletion or inhibition of Sir2 [41] to ask whether nicotinamide could inhibit the longevity effect of calorie restriction, even in the absence of Sir2. As expected, growth on 5 mM nicotinamide reduced the life span of wild-type cells to a level not significantly different from that of *sir2*Δ cells (Figure 4A). The very short life span of *sir2*Δ cells or nicotinamide-treated wild-type cells is most likely due to the hyperaccumulation of ERCs in cells lacking Sir2 activity [36,41]. Also as expected, nicotinamide had no effect on the life span of *sir2*Δ *fob1*Δ double mutants (Figure 4B), because Sir2 is already absent from these cells. CR dramatically increased the life span of *sir2*Δ *fob1*Δ double mutants, but, unexpectedly, addition of nicotinamide decreased the magnitude of life span extension conferred by CR (Figure 4B). Thus, Sir2-independent life span extension by CR is partially prevented by nicotinamide.

## Discussion

### Role of Respiration and Nicotinamide in Life Span Extension by CR

Sir2 is dispensable for life span extension by CR in yeast [36]. It remains possible, however, that under some conditions, CR might be mediated by Sir2. Central to this possibility is the premise that CR results in activation of Sir2. One mechanism by which CR has been hypothesized to activate Sir2 involves altered levels of the nicotinamide adenine dinucleotide cofactors NAD^+^ and NADH, resulting from a metabolic shift toward increased respiration in response to CR [46,47]. The other mechanism by which CR has been suggested to activate Sir2 is through a reduction in nicotinamide levels [49].

**Figure 5:**
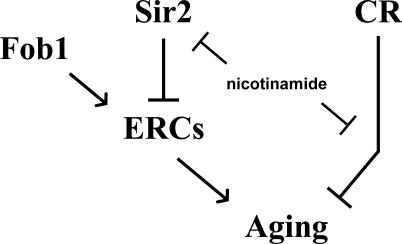
Genetic Pathways Determining Replicative Life Span in Yeast Sir2 and CR act in parallel pathways to slow aging. Both pathways are affected by nicotinamide levels.

An important test of the respiration-dependent model for CR is whether CR can increase life span in cells that are incapable of respiration. Contradictory to the prediction from this model, we find that respiration is dispensable for enhanced longevity in response to CR. Growth on reduced glucose resulted in increased life span in two distinct models of respiratory deficiency, *cyt1*Δ and rho^0^ (see Figures 1C and 2C–2E**).** These data, combined with the observation that inhibition of Sir2 cannot account for the effect of nicotinamide on life span extension by CR (Figure 4B), call into question the proposed molecular explanations for activation of Sir2 in response to CR. Further, we find no evidence that Sir2 activity is altered either by CR or by respiratory deficiency, as measured by Sir2-dependent transcriptional silencing near telomeres (see Figure 3B and 3C). This result does not rule out the possibility that CR specifically enhances Sir2 activity at the rDNA; however, like the case at telomeres, Sir2-dependent silencing of a modified *URA3* marker gene inserted into the rDNA is not enhanced by CR (J. Smith, personal communication). Thus, we propose that life span extension by CR occurs through a conserved Sir2-independent, respiration-independent mechanism (Figure 5).

It should be noted that our results do not contradict previous findings that increased respiration correlates with replicative life span in PSY316. Overexpression of the *HAP4* transcription factor, which results in transcriptional up-regulation of respiratory genes and increased oxygen consumption, or overexpression of a mitochondrial NADH oxidoreductase, are reported to increase life span in PSY316 [46,47]. It remains possible that these interventions do indeed activate Sir2 by altering levels of NAD^+^ or NADH, as proposed. Alternatively, these interventions may behave as genetic mimics of CR, increasing life span through a Sir2-independent mechanism.

Our data suggest that high levels of nicotinamide can alter the response of yeast cells to CR. How might nicotinamide interfere with life span extension by CR? We can imagine at least three possible models. First, nicotinamide could partially block CR by inhibiting the activity of the other yeast Sirtuins (Hst1–4). This model seems unlikely because CR increases the life span of yeast cells lacking both Sir2 and either Hst1, Hst2, Hst3, or Hst4, and CR increases the life span of a *sir2*Δ *fob1*Δ *hst1*Δ *hst2*Δ quadruple mutant by greater than 50% (unpublished data). Second, nicotinamide could specifically interfere with the longevity benefits of CR, but through a mechanism unrelated to Sirtuin action. Nicotinamide, conventionally classified as a vitamin, participates in many biological processes distinct from Sirtuins [59], and could conceivably alter the activity of any NAD^+^–binding protein in the cell. Third, a reduction in nicotinamide levels conferred by CR might be important to offset detrimental effects, resulting from growth on reduced glucose medium, that are themselves unrelated to replicative aging, but may shorten life span to an extent that it masks life span extension by CR. Further study will be required to distinguish between these models.

### Mitochondrial Defects, CR, and Longevity

Defects in mitochondrial function cause several human diseases, and mutation of mitochondrial DNA has been suggested to result in age-associated phenotypes in mammals [60–62]. Yeast provides a unique model in which to study the phenotypic consequences of mutation to the mitochondrial genome. With respect to replicative life span, complete deletion of the mitochondrial genome (rho^0^) results in different phenotypic outcomes depending on the genetic background of the strain [58,63]. Indeed, we report here that rho^0^ cells of BY4742 have a life span comparable to that of wild-type cells, whereas, rho^0^ cells of PSY316 are extremely short-lived (compare Figure 1C with Figure 2C). Presumably, this difference is the result of polymorphisms present in the nuclear genomes of these strains. Interestingly, in the PSY316 strain background, a nuclear mutation (*cyt1*Δ) that prevents respiration results in a life span comparable to that of wild-type cells (see Figure 2D). Thus, the short life span of PSY316 rho^0^ cells is apparently caused by loss of mitochondrial DNA rather than a general consequence of respiratory deficiency.

Although the PSY316 rho^0^ variant is extremely short-lived, CR by growth on low glucose is capable of increasing the life span of these cells by more than 100% (see Figure 3A). In fact, CR increases life span of the rho^0^ strain to a level that is comparable to calorie-restricted wild-type cells. To the best of our knowledge, this is the first indication, in any organism, that CR has a beneficial effect on defects caused by deletion of mitochondrial DNA. It will be of interest to understand the molecular basis for this effect and to determine whether this is a general feature of CR in multicellular eukaryotes.

### Conclusion

Three competing models of life span extension by CR in yeast have been put forward: (1) Sir2 activation through a metabolic shift to respiration [46,47], (2) Sir2 activation by decreased nicotinamide levels [49], and (3) Sir2-independent life span extension [28,36]. Although CR can increase life span by a Sir2-independent mechanism [36], it remains to be determined whether either of the Sir2-dependent models account for a portion of the longevity benefits of CR under any conditions. We show here that in two different strain backgrounds, one of which is the PSY316 strain background used to generate the data supporting the Sir2-dependent models, life span extension by CR does not require respiration. We also show that the partial inhibition of CR by addition of exogenous nicotinamide does not act through Sir2. Thus, activation of Sir2 through a metabolic shift to respiration or through depletion of intracellular nicotinamide cannot explain CR-mediated increases in longevity.

## Materials and Methods

### Strains and media.

Unless otherwise stated, all yeast strains were derived from the parent strain for the haploid yeast open reading frame (ORF) deletion collection [64], BY4742 (obtained from Research Genetics, Invitrogen, Carlsbad, California, United States) or from PSY316AUT [55]. Strains used in this study are listed in Table 1. Gene disruptions were carried out by transforming yeast with PCR-amplified deletion constructs containing 45 nucleotides of homology to regions flanking the ORF to be deleted and either *HIS3, LEU2,* or *URA3* amplified from pRS403, pRS405, or pRS406 [65], respectively. In each case, the entire ORF of the deleted gene was removed. All gene disruptions were verified by PCR. Medium used for life span studies was YEP (2% bacto peptone, 1% yeast extract) supplemented with filter-sterilized glucose at the designated concentration. For nicotinamide supplementation experiments, nicotinamide was added to YEP from a 500 mM nicotinamide (100×) filter sterilized stock solution to a final concentration of 5 mM just prior to pouring plates. Nicotinamide was obtained from Sigma (St. Louis, Missouri, United States).

**Table 1:**
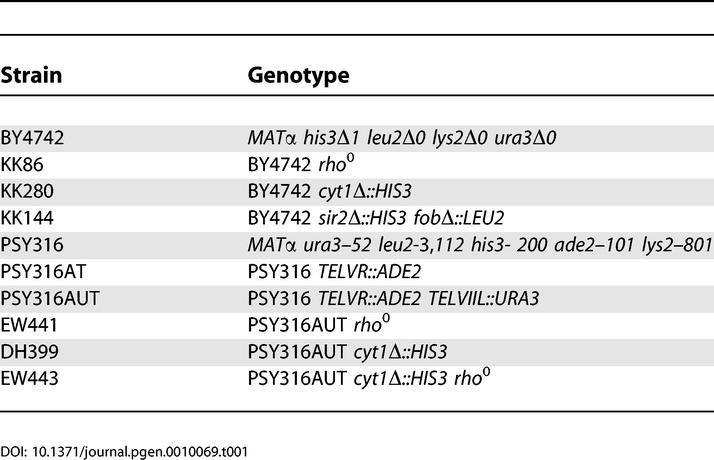
Yeast Strains Used in This Study

### Generation of rho^0^ strains and verification by DAPI staining.

The rho^0^ strains used for life span analysis were generated by treatment with ethidium bromide. In each case, life span was determined for more than one rho^0^ isolate in order to verify the observed phenotype. In the case of PSY316AUT rho^0^, four different rho^0^ isolates were examined, and the severe shortening in life span was observed in all four cases. Life span was also determined for spontaneously arising PSY316AUT rho^0^ cells, which showed a life span defect similar to that of rho^0^ cells generated by ethidium bromide. Absence of mitochondrial DNA was verified by fluorescence microscopy of log phase cells stained with DAPI.

### Replicative life span analysis.

Replicative life span analysis was carried out as described [58]. For all life span experiments, strains were coded such that the researcher performing the life span experiment had no knowledge of the strain genotypes. Unless otherwise stated, standard life span medium was YEP + 2% glucose (YPD) and CR medium was YEP + 0.05% glucose. Life span experiments in the presence of nicotinamide were carried out at a final concentration of 5 mM nicotinamide in the plates. Cells were grown on experimental medium for at least 8 h prior to microdissection. Wilcoxon *p*-values were calculated using the MATLAB “ranksum” function, and strains are stated to have a significant difference in life span for *p* < 0.05.

### FOA telomere silencing assays.

For the silencing experiment shown in Figure 3B and Figure S1, three independent cultures were inoculated from single colonies into liquid YPD for each genotype and grown overnight. The next morning, each overnight culture was diluted 1:100 into YPD or CR medium and grown for 4 h in a shaking incubator. Cultures were then diluted to a cell density of approximately 2 × 10^3^ cells/ml in water, and plated in 100-μl aliquots onto synthetic complete (SC) or FOA medium, containing either 2% or 0.05% glucose, such that cells cultured in 2% glucose were plated onto 2% glucose plates and cells cultured in CR medium were plated onto 0.05% glucose plates (CR plates). Percent survival was calculated as the number of colonies arising on FOA medium divided by the number of colonies arising on SC medium. Nicotinamide silencing experiments were carried out as above, except that after the overnight culture, cells were preincubated for 4 h in YPD + 5 mM nicotinamide and plated onto SC + 5 mM nicotinamide or FOA + 5 mM nicotinamide.

For the silencing experiment shown in Figure 3C, cultures of wild-type or *cyt1*Δ cells were inoculated from single colonies into liquid YPD or CR medium. The next morning, each overnight culture was diluted 1:1000 into fresh control or CR medium, such that cells grown overnight in control medium were diluted in control medium and cells grown overnight in CR medium were diluted into CR medium, and grown for 8 h in a shaking incubator. Cell cycle division time for BY4742 control cells was approximately 95 min and for BY4742 CR cells was approximately 105 min. After outgrowth, cultures were then diluted to a cell density of approximately 2 × 10^3^ cells/ml in water, and plated in 100-μl aliquots onto SC or FOA medium, containing either 2% or 0.05% glucose, such that cells cultured in 2% glucose were plated onto 2% glucose plates and cells cultured in CR medium were plated onto CR plates. Percent survival was calculated as the number of colonies arising on FOA medium divided by the number of colonies arising on SC medium.

## Supporting Information

Figure S1 CR Has No Effect on Sir2 Activity in Respiratory-Competent or Respiratory-Deficient Cells Transcriptional silencing of the telomeric URA3 marker in PSY316AUT was monitored by the survival of cells plated onto medium containing 5-FOA.
(42 KB PDF)

## Abbreviations

CR: calorie restriction
ERC: extrachromosomal rDNA circles
ORF: open reading frame
SC: synthetic complete

## References

[pgen-0010069-b001] Walker G, Houthoofd K, Vanfleteren JR, Gems D (2005). Dietary restriction in *C. elegans:* From rate-of-living effects to nutrient sensing pathways. Mech Ageing Dev.

[pgen-0010069-b002] Merry BJ (2005). Dietary restriction in rodents—Delayed or retarded ageing. Mech Ageing Dev.

[pgen-0010069-b003] Partridge L, Piper MDW, Mair W (2005). Dietary restriction in *Drosophila*. Mech Ageing Dev.

[pgen-0010069-b004] Sinclair DA (2005). Toward a unified theory of caloric restriction and longevity regulation. Mech Ageing Dev.

[pgen-0010069-b005] Guarente L (2005). Calorie restriction and SIR2 genes: Towards a mechanism. Mech Ageing Dev.

[pgen-0010069-b006] Merry BJ (2004). Oxidative stress and mitochondrial function with aging—The effects of calorie restriction. Aging Cell.

[pgen-0010069-b007] Armeni T, Pieri C, Marra M, Saccucci F, Principato G (1998). Studies on the life prolonging effect of food restriction: Glutathione levels and glyoxylase enzymes in rat liver. Mech Ageing Dev.

[pgen-0010069-b008] De Cabo R, Cabello R, Rios M, Lopez-Lluch G, Ingram DK (2004). Calorie restriction attenuates age-related alterations in the plasma membrane antioxidant system in rat liver. Exp Gerontol.

[pgen-0010069-b009] Guo Z, Heydari A, Richardson A (1998). Nucleotide excision repair of actively transcribed versus nontranscribed DNA in rat hepatocytes: Effect of age and dietary restriction. Exp Cell Res.

[pgen-0010069-b010] Rao KS (2003). Dietary calorie restriction, DNA repair and brain aging. Mol Cell Biochem.

[pgen-0010069-b011] Yu BP (1996). Aging and oxidative stress: Modulation by dietary restriction. Free Radic Biol Med.

[pgen-0010069-b012] Youngman LD, Park JY, Ames BN (1992). Protein oxidation associated with aging is reduced by dietary restriction of protein or calories. Proc Natl Acad Sci U S A.

[pgen-0010069-b013] Sohol RS, Weindruch R (1996). Oxidative stress, caloric restriction, aging. Science.

[pgen-0010069-b014] Masoro EJ, Katz MS, McMahan CA (1989). Evidence for the glycation hypothesis of aging from the food-restricted rodent model. J Gerontol.

[pgen-0010069-b015] Masoro EJ, McCarter RJM, Katz MS, McMahan CA (1992). Dietary restriction alters the characteristics of glucose fuel use. J Gerontol.

[pgen-0010069-b016] Kemnitz JW, Roecker EB, Weindruch R, Elson DF, Baum ST (1994). Dietary restriction increases insulin sensitivity and lowers blood glucose in rhesus monkeys. Am J Physiol.

[pgen-0010069-b017] Zhang Y, Herman B (2002). Ageing and apoptosis. Mech Ageing Dev.

[pgen-0010069-b018] Weindruch R, Walford RL (1988). The retardation of aging and disease by dietary restriction.

[pgen-0010069-b019] Jolly CA (2005). Diet manipulation and prevention of aging, cancer, and autoimmune disease. Curr Opin Clin Nutr Metab Care.

[pgen-0010069-b020] Mattson MP (2000). Emerging neuroprotective strategies for Alzheimer's disease: Dietary restriction, telomerase activation, and stem cell therapy. Exp Gerontol.

[pgen-0010069-b021] Saffrey MJ (2004). Ageing of the enteric nervous system. Mech Ageing Dev.

[pgen-0010069-b022] McCay CM, Crowell MF, Maynard LA (1935). The effect of retarded growth upon the length of life and upon ultimate size. J Nutr.

[pgen-0010069-b023] Masoro EJ (1998). Influence of caloric intake on aging and on the response to stressors. J Toxicol Environ Health B Crit Rev.

[pgen-0010069-b024] Mattson MP, Duan W, Guo Z (2003). Meal size and frequency affect neuronal plasticity and vulnerability to disease: Cellular and molecular mechanisms. J Neurochem.

[pgen-0010069-b025] Murphy CT, McCarroll SA, Bargmann CI, Fraser A, Kamath RS (2003). Genes that act downstream of DAF-16 to influence the lifespan of *Caenorhabditis elegans*. Nature.

[pgen-0010069-b026] Kenyon C (2005). The plasticity of aging: Insights from long-lived mutants. Cell.

[pgen-0010069-b027] Braeckman BP, Houthoofd K, Vanfleteren JR (2001). Insulin-like signaling, metabolism, stress resistance and aging in *Caenorhabditis elegans*. Mech Ageing Dev.

[pgen-0010069-b028] Kaeberlein M, Powers RW, Steffen KK, Westman EA, Hu D (2005). Regulation of yeast replicative life span by Tor and Sch9 in response to nutrients. Science.

[pgen-0010069-b029] Meissner B, Boll M, Daniel H, Baumeister R (2004). Deletion of the intestinal peptide transporter affects insulin and TOR signaling in *Caenorhabditis elegans*. J Biol Chem.

[pgen-0010069-b030] Vellai T, Takacs-Vellai K, Zhang Y, Kovacs AL, Orosz L (2003). Genetics: Influence of TOR kinase on lifespan in *C. elegans*. Nature.

[pgen-0010069-b031] Kapahi P, Zid BM, Harper T, Koslover D, Sapin V (2004). Regulation of lifespan in *Drosophila* by modulation of genes in the TOR signaling pathway. Curr Biol.

[pgen-0010069-b032] Sharp ZD, Bartke A (2005). Evidence for down-regulation of phosphoinositide 3-kinase/Akt/mammalian target of rapamycin (PI3K/Akt/mTOR)-dependent translation regulatory signaling pathways in Ames dwarf mice. J Gerontol A Biol Sci Med Sci.

[pgen-0010069-b033] Lin SJ, Defossez PA, Guarente L (2000). Requirement of NAD and SIR2 for life-span extension by calorie restriction in *Saccharomyces cerevisiae*. Science.

[pgen-0010069-b034] Rogina B, Helfand SL (2004). Sir2 mediates longevity in the fly through a pathway related to calorie restriction. Proc Natl Acad Sci U S A.

[pgen-0010069-b035] Guarente L, Picard F (2005). Calorie restriction—The SIR2 connection. Cell.

[pgen-0010069-b036] Kaeberlein M, Kirkland KT, Fields S, Kennedy BK (2004). Sir2-independent life span extension by calorie restriction in yeast. PLoS Biol.

[pgen-0010069-b037] Kaeberlein M, Andalis AA, Fink GR, Guarente L (2002). High osmolarity extends life span in *Saccharomyces cerevisiae* by a mechanism related to calorie restriction. Mol Cell Biol.

[pgen-0010069-b038] Smith JS, Brachmann CB, Celic I, Kenna MA, Muhammad S (2000). A phylogenetically conserved NAD^+^-dependent protein deacetylase activity in the Sir2 protein family. Proc Natl Acad Sci U S A.

[pgen-0010069-b039] Landry J, Sutton A, Tafrov ST, Heller RC, Stebbins J (2000). The silencing protein SIR2 and its homologs are NAD-dependent protein deacetylases. Proc Natl Acad Sci U S A.

[pgen-0010069-b040] Imai S, Armstrong CM, Kaeberlein M, Guarente L (2000). Transcriptional silencing and longevity protein Sir2 is an NAD-dependent histone deacetylase. Nature.

[pgen-0010069-b041] Kaeberlein M, McVey M, Guarente L (1999). The SIR2/3/4 complex and SIR2 alone promote longevity in *Saccharomyces cerevisiae* by two different mechanisms. Genes Dev.

[pgen-0010069-b042] Sinclair DA, Guarente L (1997). Extrachromosomal rDNA circles—A cause of aging in yeast. Cell.

[pgen-0010069-b043] Michel AH, Kornmann B, Dubrana K, Shore D (2005). Spontaneous rDNA copy number variation modulates Sir2 levels and epigenetic gene silencing. Genes Dev.

[pgen-0010069-b044] Kennedy BK, Austriaco NR, Zhang J, Guarente L (1995). Mutation in the silencing gene SIR4 can delay aging in *S. cerevisiae*. Cell.

[pgen-0010069-b045] Couzin J (2004). Scientific community. Aging research's family feud. Science.

[pgen-0010069-b046] Lin SJ, Kaeberlein M, Andalis AA, Sturtz LA, Defossez PD (2002). Calorie restriction extends *Saccharomyces cerevisiae* life span by increasing respiration. Nature.

[pgen-0010069-b047] Lin SJ, Ford E, Haigis M, Liszt G, Guarente L (2004). Calorie restriction extends yeast life span by lowering the level of NADH. Genes Dev.

[pgen-0010069-b048] Anderson RM, Latorre-Esteves M, Neves AR, Lavu S, Medvedik O (2003). Yeast life-span extension by calorie restriction is independent of NAD fluctuation. Science.

[pgen-0010069-b049] Anderson RM, Bitterman KJ, Wood JG, Medvedik O, Sinclair DA (2003). Nicotinamide and PNC1 govern lifespan extension by calorie restriction in *Saccharomyces cerevisiae*. Nature.

[pgen-0010069-b050] Bitterman KJ, Anderson RM, Cohen HY, Latorre-Esteves M, Sinclair DA (2002). Inhibition of silencing and accelerated aging by nicotinamide, a putative negative regulator of yeast sir2 and human SIRT1. J Biol Chem.

[pgen-0010069-b051] Landry J, Slama JT, Sternglanz R (2000). Role of NAD(+) in the deacetylase activity of the SIR2-like proteins. Biochem Biophys Res Comm.

[pgen-0010069-b052] Gallo CM, Smith DL, Smith JS (2004). Nicotinamide clearance by Pnc1 directly regulates Sir2-mediated silencing and longevity. Mol Cell Biol.

[pgen-0010069-b053] Kaeberlein M, Kennedy BK (2005). Large-scale identification in yeast of conserved ageing genes. Mech Ageing Dev.

[pgen-0010069-b054] Kaeberlein M, Andalis AA, Liszt G, Fink GR, Guarente L (2004). *Saccharomyces cerevisiae* SSD1-V confers longevity by a Sir2p-independent mechanism. Genetics.

[pgen-0010069-b055] Kaeberlein M, McDonagh T, Heltweg B, Hixon J, Westman EA (2005). Substrate-specific activation of sirtuins by resveratrol. J Biol Chem.

[pgen-0010069-b056] Tzagoloff A, Myers AM (1986). Genetics of mitochondrial biogenesis. Annu Rev Biochem.

[pgen-0010069-b057] de Zamaroczy M, Bernardi G (1986). The primary structure of the mitochondrial genome of *Saccharomyces cerevisiae*. Gene.

[pgen-0010069-b058] Kaeberlein M, Kirkland KT, Fields S, Kennedy BK (2005). Genes determining yeast replicative life span in a long-lived genetic background. Mech Ageing Dev.

[pgen-0010069-b059] Williams AC, Ramsden DB (2005). Nicotinamide homeostasis: A xenobiotic pathway that is key to development and degenerative diseases. Med Hypotheses.

[pgen-0010069-b060] Wallace DC (2001). A mitochondrial paradigm for degenerative diseases and ageing. Novartis Found Symp.

[pgen-0010069-b061] Wallace DC, Shoffner JM, Trounce I, Brown MD, Ballinger SW (1995). Mitochondrial DNA mutations in human degenerative diseases and aging. Biochim Biophys Acta.

[pgen-0010069-b062] Taylor RW, Turnbull DM (2005). Mitochondrial DNA mutations in human disease. Nat Rev Genet.

[pgen-0010069-b063] Kirchman PA, Kim S, Lai CY, Jazwinski SM (1999). Interorganelle signaling is a determinant of longevity in *Saccharomyces cerevisiae*. Genetics.

[pgen-0010069-b064] Winzeler EA, Shoemaker DD, Astromoff A, Liang H, Anderson K (1999). Functional characterization of the *S. cerevisiae* genome by gene deletion and parallel analysis. Science.

[pgen-0010069-b065] Sikorski RS, Hieter P (1989). A system of shuttle vectors and yeast host strains designed for efficient manipulation of DNA in *Saccharomyces cerevisiae*. Genetics.

