# Supplementary material for: Increased Life Span due to Calorie Restriction in Respiratory-Deficient Yeast
Source: PLoS Genet. 2005 Nov 25;1(5):e69. doi: 10.1371/journal.pgen.0010069 (PMC1287956; doi:10.1371/journal.pgen.0010069)
Supplement: Supplementary file 1 [file pgen.0010069.sg001.pdf]

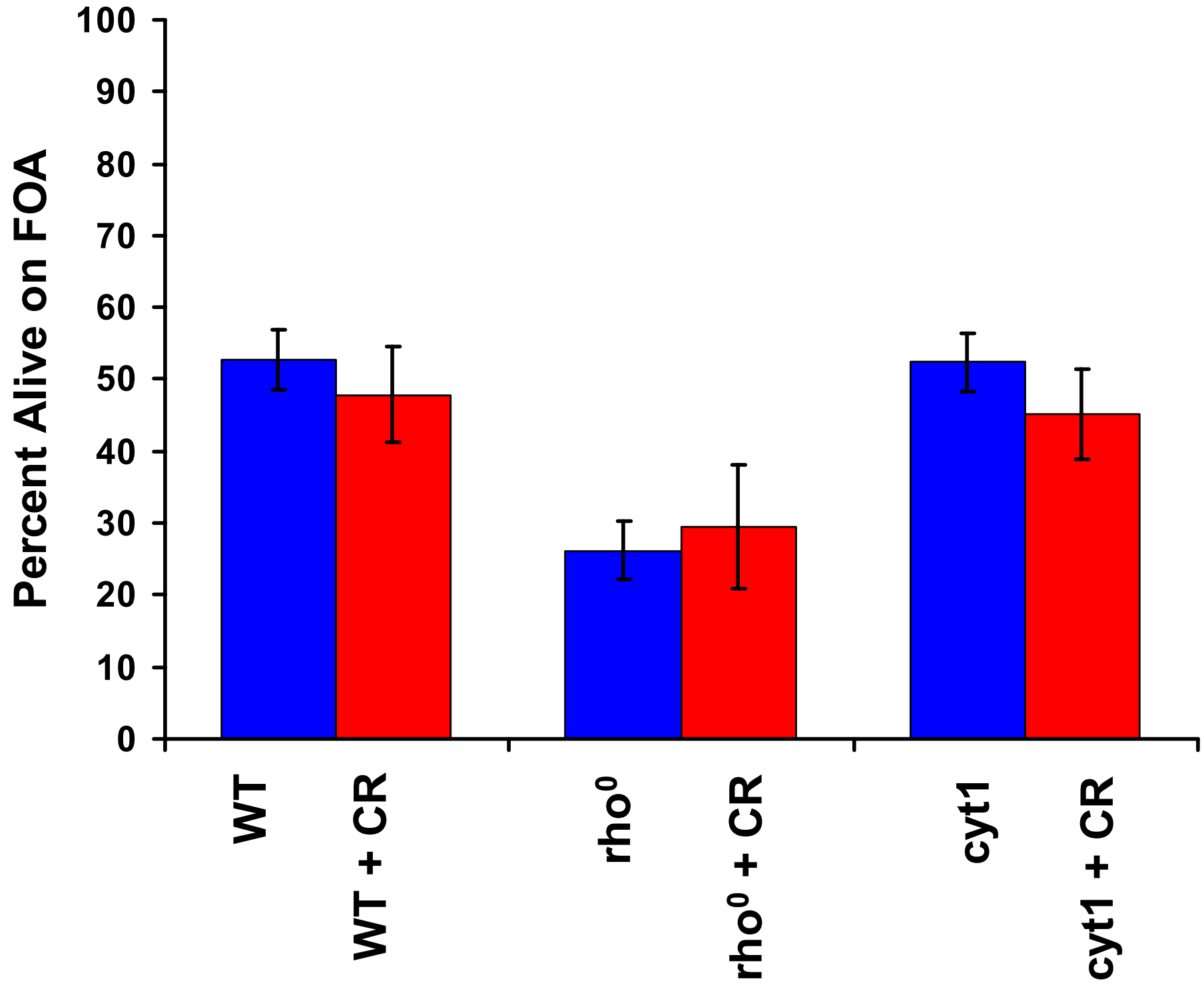

Supplemental Figure 1. CR has no effect on Sir2 activity in respiratory competent or respiratory deficient cells. Transcriptional silencing of the telomeric URA3 marker in PSY316AUT was monitored by the survival of cells plated onto media containing 5-FOA.
